# Impact of coexisting diabetes on the development of cardiovascular disease and death in patients with cancer

**DOI:** 10.1371/journal.pone.0337946

**Published:** 2026-01-22

**Authors:** Yoshihiro Kuwabara, Toshitaka Morishima, Haruka Kudo, Mizuki Shimadzu Kato, Shihoko Koyama, Kayo Nakata, Isao Miyashiro

**Affiliations:** 1 Cancer Control Center, Osaka International Cancer Institute, Osaka, Japan; 2 Department of Promoting Cooperation for Community Medicine, Graduate School of Medical Sciences, Nagoya City University, Nagoya, Japan; Ehime University Graduate School of Medicine, JAPAN

## Abstract

Diabetes is a risk factor for the development of cardiovascular disease (CVD); however, its effects on the incidence of CVD and survival in patients with cancer without CVD at the time of cancer diagnosis remain unclear. Population-based information was obtained from the Osaka Cancer Registry linked to administrative data, and the impact of diabetes at cancer diagnosis on overall survival and the development of CVD in patients without CVD at cancer diagnosis was analyzed using Cox proportional hazards models. A total of 121,997 patients diagnosed with cancer but without CVD in Osaka Prefecture, Japan, between 2010 and 2015 were included in the analysis. Of these, 4,317 patients had diabetes at the time of cancer diagnosis. The presence of coexisting diabetes was associated with increased risks of all-cause mortality (adjusted hazard ratio: 1.40 [95% confidence interval: 1.33–1.48]) and development of CVD (1.37 [1.28–1.46]), compared with the absence of coexisting diabetes. For 21,292 patients with a record of hospitalization within 2 months prior to death, the estimated cause of death was tabulated. In all, 899 (4.6%) and 111 (6.9%) patients in the no-coexistent diabetes and coexistent diabetes groups, respectively, died from circulatory disease. In patients with cancer without CVD at cancer diagnosis, coexisting diabetes may increase the risk of developing CVD and affect survival.

## Introduction

Recent advances in cancer treatment have led to improved outcomes and a remarkable increase in the number of cancer survivors [1,2]. Since cancer and cardiovascular disease (CVD) share many common risk factors and many cancer treatments increase the risk of CVD, many patients develop CVD after cancer treatment [3–5]. Therefore, the risk of CVD in patients with cancer has attracted increased attention [6]; however, factors influencing the development of CVD are not yet fully understood.

Diabetes is known to increase the risk of developing CVD in the general population [7], and improved management of diabetes is expected to improve life outcomes. However, the extent to which diabetes is associated with the risk of developing CVD and survival in patients with cancer without CVD at the time of cancer diagnosis is unknown. While the European Society of Cardiology guideline on cardio-oncology recommends appropriate management of diabetes in patients with cancer, evidence to support this recommendation remains lacking [8]. Therefore, the aim of current study was to determine the actual impact of coexisting diabetes on CVD incidence and mortality in patients with cancer.

## Methods

### Data source

In this multicenter retrospective cohort study, population-based data were collected from the Osaka Cancer Registry linked to administrative data. The Osaka Cancer Registry provides information on cancer diagnosis and survival status of patients residing in Osaka Prefecture, the third most populous prefecture in Japan, including age, sex, cancer type, cancer diagnosis date, last follow-up date, death date, and cancer stage (Surveillance, Epidemiology, and End Results [SEER] Summary Stage 2000 classification [9], such as localized, regional to lymph nodes, and regional by direct extension or distant) [10–12]. Administrative claims data were generated according to the Japanese Diagnosis Procedure Combination Per-Diem Payment System (DPC), which governs reimbursement from insurers to acute care hospitals [13,14]. DPC data, including the history of medication and clinical procedures, were collected from 36 designated cancer care hospitals in Osaka Prefecture with the support of the Council for Coordination of Designated Cancer Care Hospitals in Osaka. Designated cancer care hospitals are medical facilities certified by national or prefectural governments as having a high level of competence, experience, and leadership in cancer treatment. As of April 2024, there were 461 designated cancer care hospitals across Japan. Height, weight, and diagnosis based on the International Classification of Diseases, 10th edition (ICD-10) codes at the time of admission were recorded in the DPC data. Osaka Cancer Registry data were linked to the DPC data and anonymized thereafter [13–15]. Researchers had no access to personally identifiable data, and analyses were performed using the de-identified dataset. The researchers accessed the database for the purpose of this study on 19/04/2021.

### Study population

Patients diagnosed with cancer between 2010 and 2015 were included in the study. Patients with carcinoma in situ, a second primary cancer within two months of their first cancer diagnosis, age below 20 years or above 100 years at the time of cancer diagnosis, death certificate only, and a history of CVD at the time of first cancer diagnosis were excluded. All patients were followed up for 3 years after cancer diagnosis or until death. The median follow-up time was 36 months (interquartile range: 18.0–36.0 months).

The presence of CVD was identified using ICD-10 codes in the DPC data. The CVDs and corresponding ICD-10 codes were as follows: heart failure (HF, I50), ischemic heart disease (IHD, I20–25), peripheral arterial disease (PAD, I70–79), cerebrovascular accident (CVA, I60–69), and atrial fibrillation (Afib, I48) [16,17].

Patients were divided into two groups: with and without coexisting diabetes at the time of cancer diagnosis. The presence of diabetes was identified from the antidiabetic prescription records in the DPC data. The earliest antidiabetic prescription month was considered the time when diabetes was diagnosed. Antidiabetic medications included metformin, insulin and insulin analogs (pen-type injection device), glucagon-like peptide 1 receptor agonists, dipeptidyl peptidase 4 inhibitors, sodium glucose cotransporter 2 inhibitors, thiazolidinediones, glinides, alpha-glucosidase inhibitors, and sulfonylureas. Patients who were diagnosed with diabetes even a month after being diagnosed with cancer were included in the group without coexisting diabetes [11].

To determine whether coexisting diabetes at the time of cancer diagnosis was associated with death from CVD, the analysis was limited to cases with a record of hospitalization within 2 months prior to death. For hospitalizations within 2 months of death from the date of discharge, the ICD-10 code of the most resource-intensive diagnosis was assumed to be the cause of death, and its association with diabetes was examined.

### Statistical analysis

Baseline characteristics were presented as means and standard deviations for continuous variables and as percentages for categorical variables in each patient group.

Using the Kaplan–Meier method and log-rank test, the effect of diabetes on the survival of patients with cancer was analyzed by comparing the survival of patients with and without coexisting diabetes at the time of cancer diagnosis for a follow-up period of up to 3 years. The analysis was first applied to all cancers and then replicated for 22 specific cancer sites based on the ICD-10 classification. Next, hazard ratios (HRs) and 95% confidence intervals (CIs) were calculated using Cox proportional hazard models adjusted for age group (20–49, 50–59, 60–69, 70–79, and 80–99 years), sex, primary cancer site (analysis for all cancer sites), cancer stage at diagnosis (localized, regional to lymph nodes, regional by direct extension, distant, and unknown), and body mass index (BMI) category (< 18.5, 18.5 to 24.9, and ≥25.0 kg/m2; not measured).

The impact of coexisting diabetes on the development of CVD in patients with cancer was evaluated using Gray’s test, with death as a competing risk. The adjusted hazard ratio of coexisting diabetes for the development of CVD was calculated using a multivariate adjusted competing risk model, with death as the competing risk after adjusting for sex, age category, cancer site, cancer stage, and BMI category. Analysis was performed by CVD subtype, and cumulative incidence graphs were generated. Analysis was further performed by cancer site.

To estimate the impact of diabetes on CVD-related death in patients with cancer, those with a record of hospitalization and a discharge date close to death (within 2 months) were selected, and the ICD-10 codes of the most resources-intensive diagnosis were tabulated. Logistic regression models were used to analyze whether the coexisting diabetes was associated with reports of “Diseases of the circulatory system” (I00–I99).

As a sensitivity analysis, we also conducted each of the above analyses by excluding patients who were diagnosed with diabetes after their cancer diagnosis from the group without coexisting diabetes.

Stata SE 18 (StataCorp LLC, College Station, TX, USA) was used for statistical analysis. EZR [18] (Saitama Medical Center, Jichi Medical University, Saitama, Japan), was used to generate cumulative incidence graphs and for Gray’s test. The statistical significance level was set at α = 0.05, and all statistical analyses were two-sided.

### Ethics approval and consent to participate

This study was conducted in accordance with the ethical standards of the Declaration of Helsinki. The requirement for informed consent for participation in this study was waived in accordance with the Japanese government’s Ethical Guidelines for Medical Research Involving Human Subjects, which permits the use of an opt-out approach. Data from the Osaka Cancer Registry were obtained with permission in accordance with the Cancer Registration Promotion Act of 2013. This study protocol, including the requirement for patient consent, was approved by the Institutional Review Board of Osaka International Cancer Institute (approval number: 1707105108).

## Results

### Participant baseline characteristics

**Fig 1:**
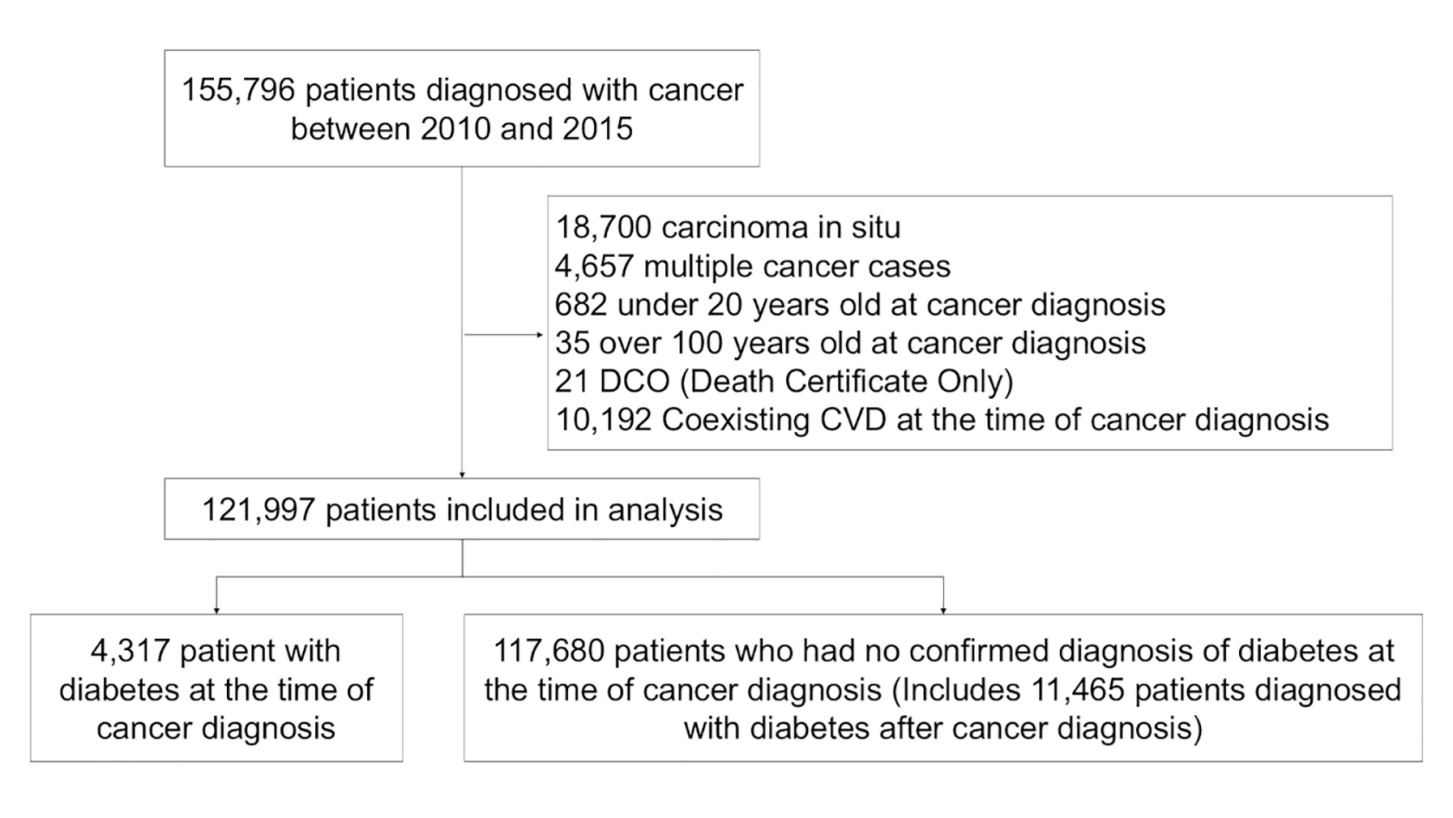
Flowchart of patients’ selection for analysis among those diagnosed with cancer between 2010 and 2015, Osaka, Japan.

Of the 121,997 patients included in the analysis, 4,317 had diabetes at the time of cancer diagnosis (Fig 1). Table 1 shows the characteristics of patients in each group. The group with coexisting diabetes consisted of a larger proportion of males (diabetes-: 56.1%, diabetes + : 67.5%), relatively older patients (diabetes-: 67.6 ± 12.5 years, diabetes + : 71.4 ± 9.3 years), and a higher proportion of patients with metastatic cancers (diabetes-: 18.9%, diabetes + : 26.6%), compared with the group without coexisting diabetes.

**Table 1:** Baseline charcteristics of patients diagnosed with cancer by diabetes status, Osaka, Japan, 2010–2015.

		Diabetes	
	Total	no	yes
n	121,997	117,680	4,317
Male, n(%)	68,910 (56.5)	65,995 (56.1)	2,915 (67.5)
Age, mean±SD	67.7 ± 12.4	67.6 ± 12.5	71.4 ± 9.3
Age category, n(%)			
20–49 years	11,963 (9.8)	11,862 (10.1)	101 (2.3)
50–59 years	13,878 (11.4)	13,555 (11.5)	323 (7.5)
60–69 years	35,627 (29.2)	34,385 (29.2)	1,242 (28.8)
70–79 years	41,394 (33.9)	39,573 (33.6)	1,821 (42.2)
80–99 years	19,135 (15.7)	18,305 (15.6)	830 (19.2)
Stage at diagnosis, n(%)			
Localized	58,071 (48.4)	57,390 (48.8)	1,681 (38.9)
Regional to lymph nodes	12,398 (10.2)	12,065 (10.3)	333 (7.7)
Regional by direct extension	19,701 (16.2)	18,889 (16.1)	812 (18.8)
Distant	23,399 (19.2)	22,252 (18.9)	1,147 (26.6)
Unknown	7,428 (6.1)	7,084 (6.0)	344 (8.0)
BMI category, n(%)			
<18.5	13,170 (10.8)	12,783 (10.9)	387 (9.0)
18.5–24.9	66,218 (54.3)	63,897 (54.3)	2,321 (53.8)
≥25.0	22,443 (18.4)	21,280 (18.1)	1,163 (26.9)
Not measured	20,166 (16.5)	19,720 (16.8)	446 (10.3)
Number of deaths in 3 years, n(%)	25,675 (21.1)	24,189 (20.6)	1,486 (34.4)
three year CVD incidence, n(%)			
HF	5,048 (4.1)	4,727 (4.0)	321 (7.4)
IHD	5,843 (4.8)	5,450 (4.6)	393 (9.1)
CVA	4,717 (3.9)	4,429 (3.8)	288 (6.7)
PAD	2,191 (1.8)	2,067 (1.8)	124 (2.9)
Afib	2,591 (2.1)	2,468 (2.1)	123 (2.9)

Patients with cardiovascular disease (CVD) at the time of cancer diagnosis are excluded. Values in parentheses represent column percentanges. ICD-10, International Classification of Diseases 10th Revision.

### Survival rates

**Fig 2:**
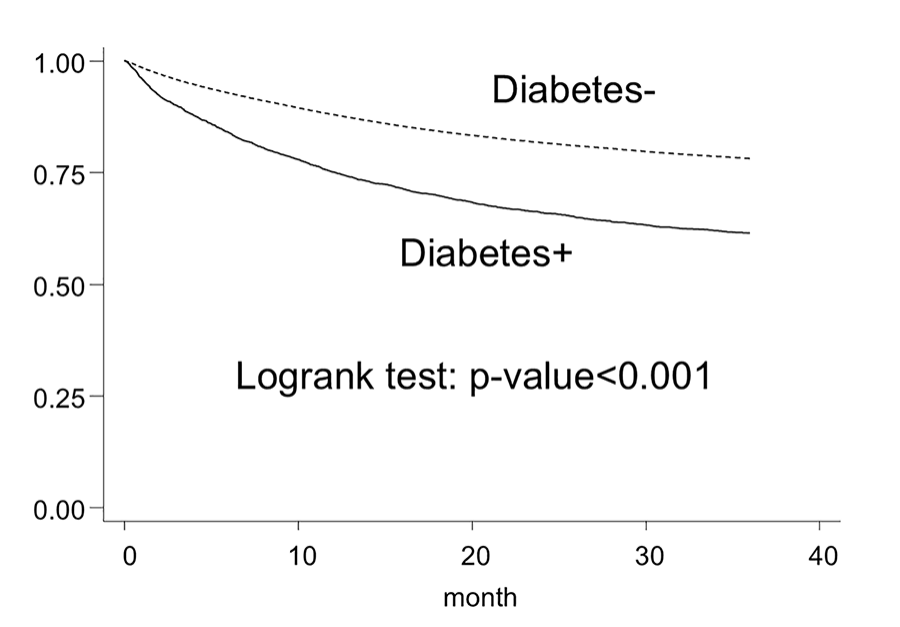
Kaplan–Meier estimates of 3-year survival among patients diagnosed with cancer between 2010 and 2015, Osaka, Japan.

The 3-year survival rate of the diabetes-free group was higher (78.2% [77.9–78.4]) than that of the diabetes group (61.5% [59.9–63.0]) (Table 2). Kaplan–Meier survival curves are shown in Fig 2. The trend toward lower survival in the diabetes group was observed in the analysis by cancer site as well; however, no difference in survival was observed for esophageal cancer. The adjusted hazard ratio (95% CI) of all-cause mortality in the group with coexisting diabetes was 1.40 (1.33–1.48), derived from the Cox proportional hazards model adjusted for sex, age category, cancer site, cancer stage, and BMI category, using the group without coexisting diabetes as reference. Analysis by cancer site showed that coexisting diabetes was associated with poor prognosis for most cancer sites, although no difference was observed between the two groups for cancers of the oral cavity/pharynx, esophagus, gallbladder, prostate, and kidney/urinary tract and leukemia (Table 3). Sensitivity analyses excluding patients who were diagnosed with diabetes after their cancer diagnosis yielded similar results.

**Table 2:** Three-year overall survival and 95% confidence interval of patients diagnosed with cancer by diabetes status, Osaka, Japan, 2010–2015.

	Diabetes			
	no		yes	
Site of first primary cancer	No. of patients (%)	3-year overall survival (95%CI)	No. of patients (%)	3-year overall survival (95%CI)
Site of Cancer based on the ICD-10 codes				
All cancer site (C00–C96)	117,680 (100)	78.2 (77.9–78.4)	4,317 (100)	61.5 (59.9–63.0)
Oral cavity/pharynx (C00–C14)	3,235 (2.75)	77.1 (75.6–78.6)	51 (1.18)	67.9 (51.5–79.8)
Esophagus (C15)	3,809 (3.24)	67.1 (65.4–68.6)	91 (2.11)	67.0 (55.2–76.4)
Stomach (C16)	16,929 (14.39)	78.7 (78.1–79.4)	612 (14.18)	75.5 (71.6–78.9)
Colorectum (C18–C20)	15,524 (13.19)	83.8 (83.1–84.3)	597 (13.83)	74.9 (71.1–78.3)
Liver (C22)	6,264 (5.32)	63.8 (62.5–65.0)	457 (10.59)	55.8 (50.7–60.5)
Gallbladder (C23–C24)	2,238 (1.90)	45.9 (43.6–48.2)	154 (3.57)	39.4 (30.3–48.4)
Pancreas (C25)	4,137 (3.52)	33.7 (32.0–35.4)	583 (13.50)	28.8 (24.4–33.3)
Larynx (C32)	1028 (0.87)	88.8 (86.6–90.6)	22 (0.51)	75.3 (50.2–89.0)
Lung (C33–C34)	13,025 (11.07)	57.2 (56.3–58.1)	528 (12.23)	39.9 (35.1–44.6)
Skin (C43–C44)	2970 (2.52)	91.3 (90.2–92.3)	48 (1.11)	77.0 (61.4–86.9)
Breast (C50)	12,030 (10.22)	95.9 (95.5–96.2)	133 (3.08)	85.8 (78.4–90.8)
Uterus (C53–C55)	4,917 (4.18)	89.7 (88.8–90.5)	79 (1.83)	84.1 (73.7–90.6)
Ovary (C56)	1,480 (1.26)	80.7 (78.5–82.6)	24 (0.56)	53.5 (30.8–71.8)
Prostate (C61)	10,728 (9.12)	94.7 (94.3–95.2)	271 (6.28)	92.0 (88.0–94.7)
Kidney/urinary tract (C64–C66, C68)	3,532 (3.00)	81.6 (80.2–82.8)	106 (2.46)	75.3 (65.4–82.8)
Bladder (C67)	2,672 (2.27)	78.2 (76.5–79.8)	79 (1.83)	65.6 (53.3–75.3)
Brain/central nervous system (C70–C72)	582 (0.49)	58.1 (53.3–62.6)	20 (0.46)	21.4 (3.5–49.4)
Thyroid (C73)	1,940 (1.65)	95.9 (94.9–96.7)	44 (1.02)	86.1 (71.6–93.5)
Malignant lymphoma (C81–C85, C96)	4,188 (3.56)	79.5 (78.3–80.8)	176 (4.08)	61.6 (53.5–68.7)
Multiple myeloma (C88–C90)	751 (0.64)	73.9 (70.5–77.0)	34 (0.79)	47.8 (29.9–63.6)
Leukemia (C91–C95)	1,572 (1.34)	55.8 (53.2–58.4)	87 (2.02)	40.0 (28.9–50.8)
Others	4,129 (3.51)	70.3 (68.9–71.8)	121 (2.80)	49.6 (39.5–58.8)

ICD-10, International Classification of Diseases 10th Revision.

**Table 3:** Adjusted hazard ratios of coexisting diabetes on all-cause mortality derived from Cox proportional hazards models according to cancer site.

Site of Cancer based on the ICD-10 codes	Adjusted HR (95% CI)	p value
All cancer site (C00–C96)	1.40 (1.33–1.48)	<0.001
Oral cavity/pharynx (C00–C14)	1.59 (0.92–2.75)	0.099
Esophagus (C15)	0.98 (0.66–1.45)	0.920
Stomach (C16)	1.26 (1.06–1.50)	0.010
Colorectum (C18–C20)	1.50 (1.26–1.78)	<0.001
Liver (C22)	1.24 (1.06–1.45)	0.006
Gallbladder (C23–C24)	1.03 (0.81–1.30)	0.813
Pancreas (C25)	1.23 (1.09–1.38)	0.001
Larynx (C32)	3.33 (1.33–8.35)	0.010
Lung (C33–C34)	1.71 (1.51–1.93)	<0.001
Skin (C43–C44)	2.81 (1.44–5.50)	0.003
Breast (C50)	2.03 (1.24–3.32)	0.005
Uterus (C53–C55)	1.96 (1.09–3.53)	0.025
Ovary (C56)	3.82 (1.99–7.32)	<0.001
Prostate (C61)	1.07 (0.69–1.65)	0.778
Kidney/urinary tract (C64–C66, C68)	0.8 (0.53–1.22)	0.297
Bladder (C67)	2.58 (1.71–3.88)	<0.001
Brain/central nervous system (C70–C72)	2.18 (1.10–4.33)	0.026
Thyroid (C73)	4.45 (1.80–11.05)	0.001
Malignant lymphoma (C81–C85, C96)	1.71 (1.32–2.22)	<0.001
Multiple myeloma (C88–C90)	2.36 (1.42–3.94)	0.001
Leukemia (C91–C95)	1.35 (0.99–1.83)	0.057
Others	1.82 (1.37–2.41)	<0.001

Hazard ratios for the patients with coexisting diabetes at cancer diagnosis are shown with those without coexisting diabetes as reference. Patients with cardiovascular disease (CVD) at the time of cancer diagnosis are excluded. All models are adjusted for age category, sex, first cancer site (at the time of analysis for all cancer sites), stage at diagnosis and body mass index category. 95% CI, 95% confidence interval; HR, hazard ratio; ICD-10, International Classification of Diseases 10th Revision.

### Cumulative incidence of cardiovascular disease

**Fig 3:**
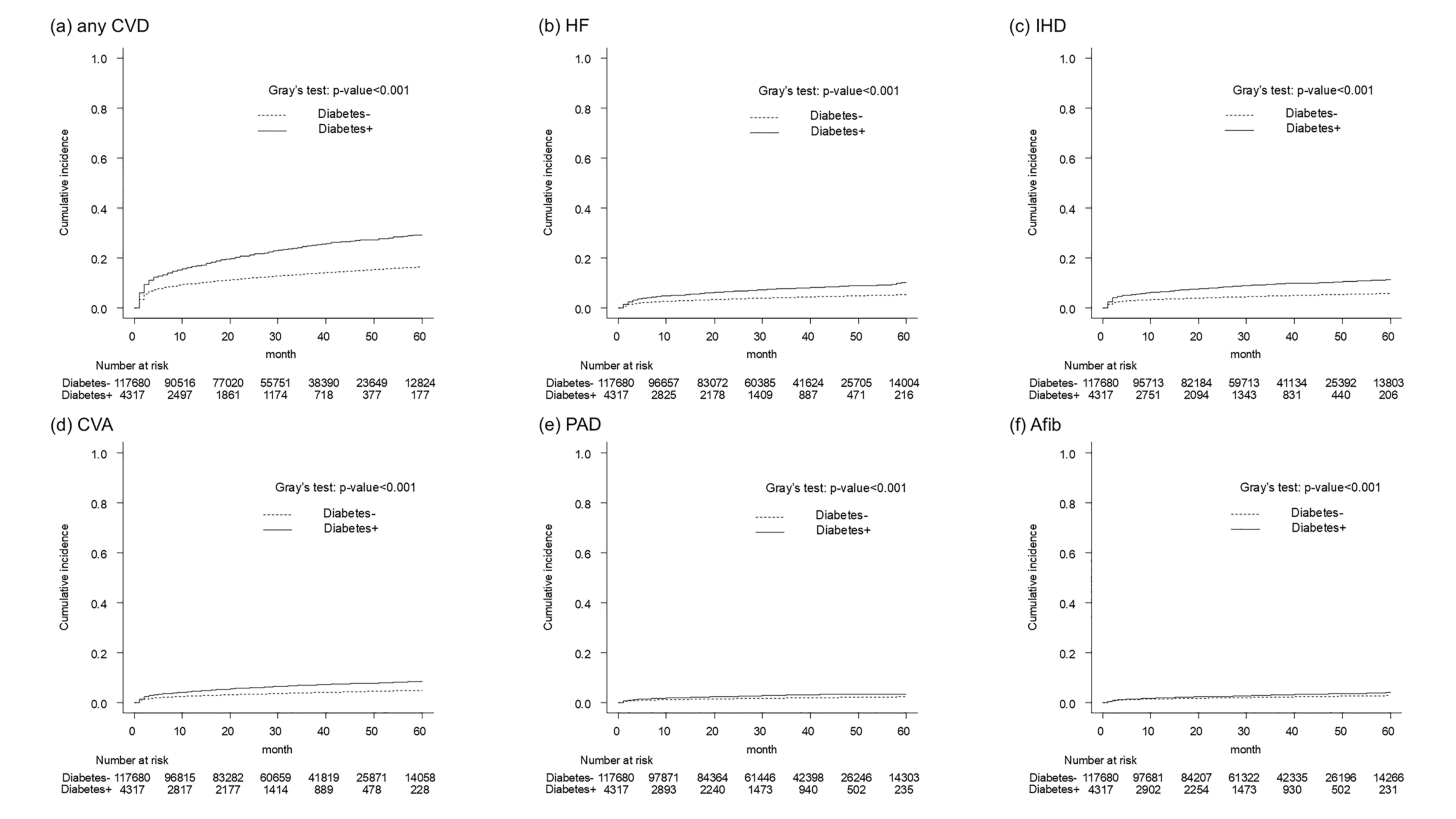
Cumulative probability of developing any CVD (a), HF (b), IHD (c), CVA (d), PAD (e), Afib (f) among patients diagnosed with cancer between 2010 and 2015, Osaka, Japan. CVD, cardiovascular diseases; HF, heart failure; IHD, ischemic heart disease; CVA, cerebrovascular accident; PAD, peripheral arterial disease; Afib, atrial fibrillation.

Gray’s test showed that the effect of coexisting diabetes on the development of each CVD was statistically significant (Fig 3a–f).

A competing risk regression analysis was performed with CVD incidence as the outcome and death as the competing risk (Table 4). The adjusted hazard ratios for coexisting diabetes were as follows: Any CVD: 1.43 (1.34–1.53), HF: 1.50 (1.34–1.67), IHD: 1.60 (1.45–1.77), CVA: 1.38 (1.23–1.55), PAD: 1.29 (1.08–1.54), and Afib: 1.08 (0.91–1.28). In the analysis according to cancer site, the risk of developing CVD was higher in patients with cancers of the stomach, colorectum, liver, gallbladder, lung, breast, prostate, kidney/urinary tract, and bladder. In addition, the risk of developing HF, IHD, CVA, and PAD varied by cancer site. No clear association was observed between coexisting diabetes and the risk of developing Afib. Sensitivity analyses excluding patients who were diagnosed with diabetes after their cancer diagnosis yielded similar results.

**Table 4:** Adjusted hazard ratios and 95% confidence intervals of coexisting diabetes on incidence of each CVD subtypes evaluated by multivariable logistic regression models according to cancer site.

	Any CVD		HF		IHD		CVA		PAD		Afib	
Site of Cancer based on the ICD-10 codes	HR (95%CI)	p value	HR (95%CI)	p value	HR (95%CI)	p value	HR (95%CI)	p value	HR (95%CI)	p value	HR (95%CI)	p value
All cancer site (C00–C96)	1.43 (1.34–1.53)	<0.001	1.50 (1.34–1.67)	<0.001	1.60 (1.45–1.77)	<0.001	1.38 (1.23–1.55)	<0.001	1.29 (1.08–1.54)	0.006	1.08 (0.91–1.28)	0.392
Oral cavity/pharynx (C00–C14)	0.47 (0.17–1.25)	0.13	0.91 (0.22–3.71)	0.892	0.3 (0.04–2.15)	0.231	NA		0.95 (0.13–6.7)	0.957	0.77 (0.11–5.4)	0.790
Esophagus (C15)	0.96 (0.61–1.51)	0.874	0.74 (0.3–1.81)	0.51	1.05 (0.49–2.25)	0.904	1.28 (0.57–2.85)	0.552	1 (0.31–3.23)	0.995	0.73 (0.27–2)	0.543
Stomach (C16)	1.79 (1.54–2.07)	<0.001	1.33 (0.96–1.83)	0.086	2.13 (1.74–2.61)	<0.001	1.79 (1.37–2.33)	<0.001	1.84 (1.26–2.68)	0.002	1.29 (0.87–1.93)	0.210
Colorectum (C18–C20)	1.62 (1.38–1.91)	<0.001	2.16 (1.67–2.79)	<0.001	1.82 (1.43–2.31)	<0.001	1.24 (0.89–1.72)	0.208	1.67 (1.08–2.59)	0.021	1.3 (0.88–1.94)	0.190
Liver (C22)	1.23 (1.01–1.48)	0.037	1.29 (0.94–1.76)	0.109	1.37 (1–1.88)	0.053	1 (0.67–1.47)	0.989	0.67 (0.37–1.19)	0.172	1.25 (0.77–2.04)	0.360
Gallbladder (C23–C24)	1.52 (1.07–2.16)	0.02	1.35 (0.69–2.63)	0.378	1.75 (1–3.08)	0.051	1.6 (0.88–2.9)	0.121	0.31 (0.04–2.34)	0.256	0.81 (0.25–2.61)	0.721
Pancreas (C25)	1.06 (0.85–1.33)	0.604	1.05 (0.69–1.59)	0.832	1.08 (0.73–1.59)	0.706	1.05 (0.72–1.53)	0.799	1.16 (0.58–2.29)	0.678	1.12 (0.63–2)	0.698
Larynx (C32)	2.01 (0.80–5.01)	0.136	2.82 (0.73–10.83)	0.131	3.65 (1.22–10.89)	0.02	6.24 (2.31–16.91)	<0.001	2.61 (0.35–19.42)	0.35	NA	
Lung (C33–C34)	1.34 (1.12–1.60)	0.002	1.46 (1.08–1.97)	0.013	1.26 (0.93–1.72)	0.135	1.41 (1.04–1.91)	0.029	0.96 (0.55–1.67)	0.872	0.98 (0.61–1.56)	0.932
Skin (C43–C44)	1.49 (0.81–2.72)	0.199	1.45 (0.45–4.68)	0.538	0.63 (0.17–2.39)	0.497	1.95 (0.81–4.71)	0.137	3.25 (1.04–10.18)	0.043	1.97 (0.6–6.43)	0.260
Breast (C50)	2.00 (1.38–2.92)	<0.001	3.03 (1.77–5.18)	<0.001	1.66 (0.87–3.14)	0.122	2.83 (1.5–5.31)	0.001	0.63 (0.09–4.62)	0.649	1.72 (0.65–4.5)	0.273
Uterus (C53–C55)	1.47 (0.73–2.97)	0.284	1.71 (0.53–5.49)	0.366	0.79 (0.19–3.31)	0.75	2.31 (0.79–6.72)	0.126	0.87 (0.11–6.85)	0.891	NA	
Ovary (C56)	1.05 (0.32–3.48)	0.936	NA		1.04 (0.14–7.93)	0.967	1.02 (0.13–7.73)	0.985	2.09 (0.26–16.56)	0.485	NA	
Prostate (C61)	2.17 (1.69–2.79)	<0.001	2.65 (1.75–4.02)	<0.001	2.69 (1.92–3.77)	<0.001	1.96 (1.27–3.04)	0.002	1.87 (0.98–3.57)	0.058	1.14 (0.5–2.58)	0.751
Kidney/urinary tract (C64–C66, C68)	1.77 (1.27–2.46)	0.001	1.83 (1.04–3.22)	0.035	1.7 (1–2.92)	0.052	1.32 (0.66–2.64)	0.431	3.94 (2.11–7.36)	<0.001	0.49 (0.12–2.03)	0.327
Bladder (C67)	1.70 (1.17–2.48)	0.005	1.69 (0.84–3.44)	0.144	1.15 (0.57–2.3)	0.694	1.54 (0.75–3.16)	0.237	2.74 (1.27–5.9)	0.010	0.3 (0.04–2.11)	0.225
Brain/central nervous system (C70–C72)	1.03 (0.24–4.37)	0.967	2.04 (0.22–19.05)	0.532	3.41 (0.8–14.59)	0.099	0.67 (0.08–5.5)	0.713	NA		NA	
Thyroid (C73)	1.35 (0.67–2.70)	0.403	0.95 (0.23–3.89)	0.943	1.28 (0.38–4.25)	0.688	2.26 (0.92–5.55)	0.076	NA		NA	
Malignant lymphoma (C81–C85, C96)	1.06 (0.73–1.53)	0.769	1.04 (0.61–1.79)	0.883	0.74 (0.33–1.69)	0.478	1.59 (0.91–2.8)	0.104	0.57 (0.14–2.27)	0.422	0.42 (0.1–1.69)	0.219
Multiple myeloma (C88–C90)	1.57 (0.92–2.66)	0.097	2.36 (1.16–4.82)	0.018	2.03 (0.92–4.47)	0.079	0.29 (0.04–2.15)	0.227	1.61 (0.39–6.74)	0.511	1.53 (0.34–6.85)	0.578
Leukemia (C91–C95)	0.90 (0.51–1.58)	0.704	0.32 (0.1–1.005)	0.05	1.9 (0.77–4.74)	0.166	1.63 (0.69–3.84)	0.265	NA		NA	
Others	1.27 (0.86–1.85)	0.228	2.17 (1.34–3.49)	0.002	1.58 (0.86–2.9)	0.137	0.45 (0.14–1.44)	0.179	0.36 (0.05–2.64)	0.315	1.41 (0.57–3.48)	0.453

### Association between coexisting diabetes and cause of death

Table 5 shows the estimated causes of death based on ICD-10 codes of 21,292 patients with a record of hospitalization close to the time of death (within two months) divided into groups with and without coexisting diabetes. The rate of death from CVD (ICD-10 codes, I00–I99) was higher in the group with coexisting diabetes than in the group without coexisting diabetes (6.94% vs. 4.57%). Logistic regression analysis adjusted for age, sex, BMI category, cancer stage, and cancer site showed a significant association between coexisting diabetes and death from CVD (ICD-10 code I00–I99 disease; odds ratio 1.47, [95% CI 1.19–1.81]) (Table 6). In the analysis according to cancer site, coexisting diabetes significantly increased the odds of CVD-related death in patients with cancers of the stomach and gallbladder (Table 6).

**Table 5:** Number of patients by cause of death estimated from the most resource-intensive diagnoses in the hospitalization where the date of death was within two months of discharge.

ICD10 codes	Diabetes		total No. of patients (%)
	no	yes	
	No. of patients (%)	No. of patients (%)	
A00-B99 Certain infectious and parasitic diseases	649 (3.3)	58 (3.6)	707 (3.3)
C00-D49 Neoplasms	14491 (73.6)	1058 (66.1)	15549 (73.0)
D50-D89 Diseases of the blood and blood-forming organs and certain disorders involving the immune mechanism	599 (3.0)	60 (3.8)	659 (3.1)
E00-E89 Endocrine, nutritional and metabolic diseases	115 (0.6)	20 (1.3)	135 (0.6)
F01-F99 Mental, Behavioral and Neurodevelopmental disorders	6 (0.0)	0 (0.0)	6 (0.0)
G00-G99 Diseases of the nervous system	64 (0.3)	8 (0.5)	72 (0.3)
H00-H59 Diseases of the eye and adnexa H60-H95 Diseases of the ear and mastoid process	9 (0.0)	3 (0.2)	12 (0.1)
I00-I99 Diseases of the circulatory system	899 (4.6)	111 (6.9)	1010 (4.7)
J00-J99 Diseases of the respiratory system	1312 (6.7)	132 (8.3)	1444 (6.8)
K00-K95 Diseases of the digestive system	1090 (5.5)	93 (5.8)	1183 (5.6)
L00-L99 Diseases of the skin and subcutaneous tissue	18 (0.1)	3 (0.2)	21 (0.1)
M00-M99 Diseases of the musculoskeletal system and connective tissue	56 (0.3)	5 (0.3)	61 (0.3)
N00-N99 Diseases of the genitourinary system	203 (1.0)	32 (2.0)	235 (1.1)
Q00-Q99 Congenital malformations, deformations and chromosomal abnormalities	1 (0.0)	0 (0.0)	1 (0.0)
R00-R99 Symptoms, signs and abnormal clinical and laboratory findings, not elsewhere classified	16 (0.1)	0 (0.0)	16 (0.1)
S00-T88 Injury, poisoning and certain other consequences of external causes.	164 (0.8)	17 (1.1)	181 (0.9)
total	19692 (100.0)	1600 (100.0)	21292 (100.0)

**Table 6:** Odds ratios by cause of death estimated from the most resource-intensive diagnosis among hospitalizations where the date of death was within 2 months of discharge.

	Diabetes		
	no	yes	
Site of Cancer based on the ICD-10 codes	Odds ratios (95%CI)	Odds ratios (95%CI)	p value
All cancer site (C00–C96)	1 (reference)	1.47 (1.19–1.81)	<0.001
Oral cavity/pharynx (C00–C14)	1 (reference)	NA	
Esophagus (C15)	1 (reference)	2.32 (0.45–12.01)	0.315
Stomach (C16)	1 (reference)	2.16 (1.28–3.65)	0.004
Colorectum (C18–C20)	1 (reference)	1.12 (0.56–2.22)	0.747
Liver (C22)	1 (reference)	1.43 (0.86–2.40)	0.172
Gallbladder (C23–C24)	1 (reference)	2.84 (1.09–7.38)	0.032
Pancreas (C25)	1 (reference)	1.17 (0.61–2.23)	0.636
Larynx (C32)	1 (reference)	1.85 (0.10–34.68)	0.682
Lung (C33–C34)	1 (reference)	1.40 (0.83–2.36)	0.206
Skin (C43–C44)	1 (reference)	0.52 (0.04–6.33)	0.608
Breast (C50)	1 (reference)	2.27 (0.50–10.29)	0.288
Uterus (C53–C55)	1 (reference)	NA	
Ovary (C56)	1 (reference)	19.72 (0.99–391.71)	0.051
Prostate (C61)	1 (reference)	1.35 (0.47–3.85)	0.576
Kidney/urinary tract (C64–C66, C68)	1 (reference)	2.53 (0.75–8.55)	0.136
Bladder (C67)	1 (reference)	0.60 (0.07–4.81)	0.629
Brain/central nervous system (C70–C72)	1 (reference)	NA	
Thyroid (C73)	1 (reference)	NA	
Malignant lymphoma (C81–C85, C96)	1 (reference)	0.31 (0.04–2.38)	0.261
Multiple myeloma (C88–C90)	1 (reference)	0.88 (0.09–8.35)	0.913
Leukemia (C91–C95)	1 (reference)	NA	
Others	1 (reference)	1.61 (0.65–3.99)	0.307

## Discussion

The main findings of our study are as follows. First, in patients with cancer without CVD at diagnosis, coexisting diabetes was associated with poor survival, even after adjustment for various confounding factors. Second, coexisting diabetes was associated with a high risk of developing various types of CVD after cancer diagnosis. Third, coexisting diabetes was associated with death from diseases of the circulatory system.

Although diabetes has been reported to be a prognostic factor for poor survival in patients with cancer [19–21], the mechanisms by which diabetes worsens the prognosis are yet to be clarified. The possible mechanisms include advanced cancer stages in patients with diabetes [22], low socioeconomic status [23], high risk of postoperative mortality after cancer surgery [24], high risk of chemotherapy-related toxicity [25], diabetes-related impaired renal function [26], limited cancer treatment options [22], increased cardiovascular complications [27,28], and poorly controlled diabetes [29,30]. We focused on the diabetes-induced increase in CVD among the proposed mechanisms. Although interest in the increase risk in CVD and its prognostic impact on patients with cancer as a result of cancer and cancer treatment has been increasing [8], the factors that influence the development of CVD in patients with cancer are still poorly understood. Our results provide evidence supporting the impact of the mechanism by which coexisting diabetes in patients with cancer may increase their risk for developing CVD and worsen their prognosis.

The increased risk of developing CVD due to diabetes is well established in the general population. In a meta-analysis of 698,782 patients from 102 prospective studies in the general population, the adjusted HR for diabetes was 2.00 (95% CI 1.83–2.19) for coronary heart disease, 2.27 (1.95–2.65) for ischemic stroke, 1.56 (1.19–2.05) for hemorrhagic stroke, and 1.84 (1.59–2.13) for unclassifiable stroke [7]. The HR in our study was lower, possibly because death from cancer was a competing risk for developing CVD in patients with cancer [31], and the risk of developing CVD may be increased by cancer itself or by cancer treatment [4], masking the effects of diabetes.

The association between diabetes-related complications and risk of developing CVD, or the fact that the association varies by cancer location, may be influenced by differences in cancer type and cancer treatment. Although the use of human epidermal growth factor receptor-2 (HER2) inhibitors is a frequent cause of cardiac dysfunction in breast cancer, the results indicate that HER2 inhibitor therapy and diabetes interact to potentiate their effects on cardiac dysfunction. HER2 inhibition is reportedly associated with worsening diabetes [32], which is an issue requiring further investigation.

In patient groups with esophageal cancer, leukemia, and malignant lymphoma, no increase in CVD risk due to the coexistence of diabetes was observed. Qin et al. reported that diabetes was a risk factor for late-onset anthracycline-induced heart failure (AIHF) but was not associated with early-onset AIHF [33]. The lack of increased CVD risk observed in patients with these cancers in our data may be due to the insufficient follow-up period of 3 years after cancer diagnosis.

In patients with prostate cancer, the coexistence of diabetes was associated with an increased risk of developing any CVD, HF, IHD, and CVA. Since prostate cancer is a malignancy with a relatively favorable prognosis and limited impact on survival duration, the impact of CVD is thought to be greater on the prognosis of prostate cancer than on that of other cancers. Previous reports indicate that patients with diabetes have a low risk of developing prostate cancer [34,35], and diabetes is associated with increased overall mortality and non-prostate cancer-related mortality in patients with prostate cancer, but not with increase in prostate cancer-specific mortality [36]. Based on our data, it is possible that the increase in CVD contributes to non-prostate cancer-related mortality in patients with prostate cancer and diabetes. However, a clear increase in cardiovascular-related mortality was not observed in our data, which may be due to the small number of CVD events or the limited patient population.

No increase in CVD risk was observed in patients with pancreatic cancer, possibly owing to their poor prognosis, which often leads to death before CVD develops. In addition, no increase in CVD-related mortality risk was observed. However, the HR for overall mortality increased with the coexistence of diabetes. In patients with gallbladder and bile duct cancers, which also have poor prognoses, overall mortality did not increase due to diabetes; however, the HR for the occurrence of any CVD and odds ratio for CVD-related mortality increased.

Diabetes can be improved with treatment, and enhanced management may lead to a reduction in the risk of CVD and an improvement in survival prognosis, as suggested by clinical studies involving patients with type 1 diabetes [37]. However, in general, prognosis is poorer in patients with cancer than in those without cancer, and severe outcomes may occur before the effects of treating coexisting diabetes become evident. Consequently, it remains uncertain whether therapeutic interventions for diabetes are effective in this population. To clarify whether diabetes management improves prognosis in patients with coexisting cancer and diabetes, further research, such as intervention studies, is warranted.

This study has some limitations. First, information on coexisting diabetes was extracted from DPC drug prescription records, and information on preexisting CVD was derived from disease code in the DPC; DPC data were obtained only from participating hospitals, diagnostic and prescription information from other hospitals were not available. Consequently, some patients who originally had coexisting diabetes at the time of cancer diagnosis could be incorrectly classified into the group of patients without coexisting diabetes, resulting in an underestimation of the prevalence of coexisting diabetes at the time of cancer diagnosis. To address this concern, we conducted a sensitivity analysis excluding patients whose diabetes was newly confirmed after the cancer diagnosis, and the results remained consistent. Thus, we believe that we have obtained some reasonable estimates of the impact of coexisting diabetes on outcomes. Similarly, the diagnosis of CVD was also not based on clinical confirmation and misclassification may occur. Second, this study is a retrospective observational study and cannot establish a causal relationship between coexisting diabetes and the incidence of CVD. Third, some confounding factors were not accounted for in this study and would require further investigation. In particular, data on diabetes severity and glycemic control were unavailable. In addition, while various cancer treatments (e.g., radiation, chemotherapy, hormonal treatments) are known to increase CVD risk, and the presence of diabetes may potentially exacerbate this effect, these factors were not considered. Other variables, such as smoking status, physical activity, nutritional status, socioeconomic factors, non-diabetes-related complications, and type of diabetes treatment may also have influenced the outcomes, but there were not considered either. Forth, the analysis of cause of death in patients with cancer was based on the diagnosis at admission, close to the time of death; hence, only a subset of patients could be analyzed. The cause of death classification was based on DPC data rather than clinical confirmation, leaving the possibility of inaccuracy. Finally, the data in this study had a limited follow-up period, which was set to a maximum of 3 years to account for dropouts. The risk of developing CVD due to diabetes is thought to persist over the long term, and its impact may be underestimated within the analysis period of this study.

In conclusion, among patients with cancer and uncomplicated CVD at the time of cancer diagnosis, diabetes may be associated with the development of subsequent CVD development and poor prognosis. Further research, including long-term prospective studies based on detailed clinical data, is warranted to determine whether appropriate diagnosis and management of diabetes may improve the outcomes in patients with cancer.

## Supporting information

S1 Table Adjusted hazard ratios of coexisting diabetes on all-cause mortality derived from Cox proportional hazards models according to cancer site. Hazard ratios for the patients with coexisting diabetes at cancer diagnosis are shown with those without coexisting diabetes as reference. Patients diagnosed with diabetes after their cancer diagnosis or who had cardiovascular disease (CVD) at the time of cancer diagnosis are excluded. All models are adjusted for age category, sex, first cancer site (at the time of analysis for all cancer sites), stage at diagnosis and body mass index category. 95% CI, 95% confidence interval; HR, hazard ratio; ICD-10, International Classification of Diseases 10th Revision.
(XLSX)

S2 Table Adjusted hazard ratios and 95% confidence intervals of coexisting diabetes on incidence of each CVD subtypes evaluated by multivariable logistic regression models according to cancer site. Patients diagnosed with diabetes after their cancer diagnosis or who had cardiovascular disease (CVD) at the time of cancer diagnosis are excluded. All models are adjusted for age category, sex, first cancer site (at the time of analysis for all cancer sites), stage at diagnosis and body mass index category. CVD, cardiovascular disease; HF, heart failure; IHD, ischemic heart disease; CVA, CVA, cerebravascular accident; PAD, peripheral arterial disease; Afib, atrial fibrillation; ICD-10, International Classification of Diseases 10th revision.
(XLSX)
